# Intravenous versus topical tranexamic acid in lumbar interbody fusion: A protocol of randomized controlled trial

**DOI:** 10.1097/MD.0000000000020619

**Published:** 2020-06-12

**Authors:** Fei Song, Zhouhai Zheng

**Affiliations:** Department of Orthopedics, People's Hospital of Nanchuan District, Chongqing, China.

**Keywords:** lumbar interbody fusion, prospective, protocol, tranexamic acid

## Abstract

**Background:**

Questions still remain about the safest and most effective route of administration for tranexamic acid (TXA) in lumbar interbody fusion. As such, the goal of this randomized clinical trial was to assess the efficacy and safety of topical TXA compared with intravenous TXA in lumbar interbody fusion.

**Methods:**

This was a prospectively randomized trial that investigated the effectiveness and safety of the intravenous and topical administrations of TXA with regard to lumbar interbody fusion. Approval from Clinical Studies Ethical Committee in our hospital was obtained. The patients were randomized to 1 of 2 treatment options:

(1) topical group and
(2) intravenous group.

Patients, surgeons, anesthesiologists, nurses, and research assistants collecting data were blinded to group allocation. The primary outcome measures were perioperative calculated blood loss, total drain output at 24 hours, and perioperative blood transfusion rate. Secondary outcomes included an analysis of complications, namely symptomatic venous thromboembolism, cerebrovascular accident, and arterio-occlusive events. Data were analyzed using the statistical software package SPSS version 25.0 (Chicago, IL).

**Results:**

There are several limitations to this study. We did not include a group of patients who did not receive TXA. Another potential limitation is that the study population contains heterogeneity such as varying patient diagnosis and surgical technique/approach. Despite these limitations, the validity of our results should be maintained, as the same methodology was applied to both treatment arms.

**Trial registration:**

This study protocol was registered in Research Registry (researchregistry5564).

## 1 Introduction

Lumbar interbody fusion has been associated with substantial blood loss and risk of transfusion. Postoperative anemia will impede physical functioning, delay rehabilitation, and increase mortality.^[1]^ As a result, approximately one-thirds of the patients may require allogeneic blood transfusion. However, allogeneic transfusion is associated with risks for disease transmission, immunosuppression, and transfusion reactions.^[2]^

Recently, the use of tranexamic acid (TXA), a lysine analog and antifibrinolytic agent, has become more common. Surgical trauma causes hyperfibrinolysis, which induces fibrin clot dissolution to sustain bleeding.^[3]^ TXA act as a lysine analog which inhibits hyperfibrinolysis by blocking the interaction of plasminogen with fibrin to prevent the dissolution of the fibrin clot and thereby reduce bleeding.^[3–7]^ Many studies have confirmed that TXA has an effective hemostatic function in joint-replacement surgery.^[8–12]^ TXA application is relatively late for lumbar interbody fusion, which requires additional study in many aspects.

Many published studies have reported that the intravenous or topical administration of TXA plays a role in reducing the blood loss and blood transfusion rates during the perioperative period of posterior lumbar interbody fusion.^[1,13]^ However, questions still remain about the safest and most effective route of administration. If the drug is administered systemically, thrombosis may be a concern in certain populations. Therefore, some practitioners advocate topical application of TXA in the surgical wound. However, the efficacy and safety of topical TXA have not been well reported. As such, the goal of this randomized clinical trial was to assess the efficacy and safety of topical TXA compared with intravenous TXA in lumbar interbody fusion.

## 2 Materials and methods

### 2.1 Study design

**Figure 1:**
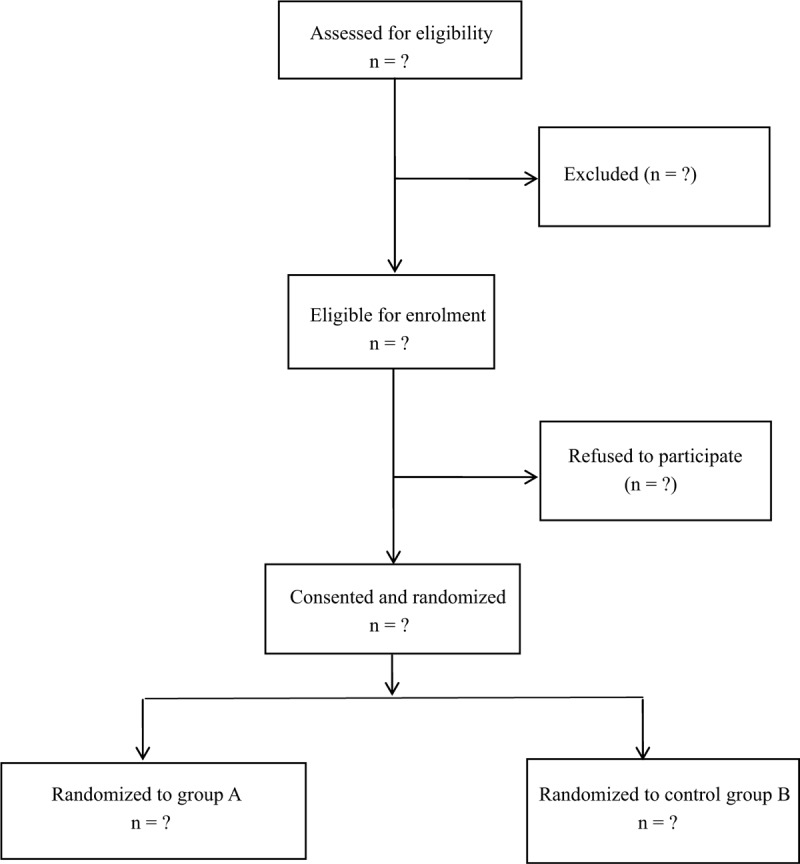
Flow diagram of the study.

This was a prospectively randomized trial that investigated the effectiveness and safety of the intravenous and topical administrations of TXA with regard to lumbar interbody fusion. Approval from Clinical Studies Ethical Committee in our hospital was obtained. This study has been published at the Research Registry (researchregistry5564). We followed the Consolidated Standards of Reporting Trials guidelines for reporting randomized trials and provided a consolidated standards of reporting trials flow diagram (Fig. 1).

### 2.2 Patients

Patients diagnosed with lumbar degenerative disease at our hospital and who had no history of posterior lumbar decompression or interbody fusion with pedicle screw fixation were selected for this study. Before surgery, informed consents were obtained from all patients after a full explanation of the therapeutic procedure.

The exclusion criteria were as follows:

(1) history of thromboembolism or evidence of existing thrombus on preoperative vascular B-mode ultrasound;
(2) use of antiplatelet aggregation drugs within 6 months or symptom of coagulation dysfunction before surgery;
(3) internal diseases such as cardiovascular disease, hepatorenal insufficiency, and hematologic system disease;
(4) confirmed allergy history or high risk of allergy to TXA;
(5) history of smoking (more than 10 cigarettes per day for more than 6 months) or drinking (at least 50 g of liquor with an alcohol volume ratio over 40% per day for more than 3 months) with unsuccessful cessation within 6 months before surgery; 6) a body mass index less than 18.5 or over 30.0; and 7) an inability to understand the study protocol after explanation or an unwillingness to participate.

### 2.3 Randomization and blinding

The patients were randomized to 1 of 2 treatment options: A) topical group and B) intravenous group. Randomization was performed without any stratification. Randomization listings were prepared with a probability of 0.4 to 0.6 and after that, randomization letters were printed according to the results of the randomization. After the patient had given consent, a member of the in-hospital clinical study center chose 1 of the 2 letters and the patient was assigned to 1 group. Patients, surgeons, anesthesiologists, nurses, and research assistants collecting data were blinded to group allocation.

### 2.4 Surgical techniques and rehabilitation exercise

All operations were performed using the same surgical technique. After performing posterior decompression, 2 polyetheretherketone cages for interbody fusion and posterior stabilization with pedicle screws and rods were utilized in all patients. To improve bone fusion, a mixture of a locally-harvested autograft obtained during posterior decompression and a demineralized bone matrix was packed inside and outside the polyetheretherketone cages. All patients were managed with the same postoperative medications and rehabiliation program protocols. Patients wore a lumbo-sacral orthosis for 3 months after the surgery and were allowed to ambulate on the first day post-surgery. Patients were not permitted to sit for long periods of time for the first month after surgery, and at 3 months post-surgery, patients were allowed to resume normal activities.rehabilitation exercise.

### 2.5 Interventions

For patients in the intravenous group, the TXA (15 mg/kg dissolved in 100 mL of normal saline) was started 30 minutes before surgery and completed 15 minutes before surgery. During surgery, the intravenous administration of TXA was maintained at a dose of 1 mg/kg, and 4 pieces of gelatin sponges soaked in 50 mL of saline for 5 minutes were placed in the surgical area before incision closure. For the topical group, 100 mL of normal saline was administered intravenously 30 minutes before surgery, and 4 pieces of gelatin sponge saturated with TXA (1 g TXA dissolved in 50 mL of normal saline and gelatin sponge soaked therein for 5 minutes) were placed flat in the surgical area before incision closure. A standard closed suction drain was placed before the wound was closed. All drains were removed 24 hours after placement.

### 2.6 Outcome measures

The primary outcome measures were perioperative calculated blood loss, total drain output at 24 hours, and perioperative blood transfusion rate. The calculated blood loss was determined from the difference between the preoperative hemoglobin level and the lowest postoperative hemoglobin level during the hospital stay (or prior to transfusion, if applicable) according to the formula by Nadler et al. Of note, drain output is not accounted for in this calculation. Other, secondary outcomes included an analysis of complications, namely symptomatic venous thromboembolism, cerebrovascular accident, and arterio-occlusive events (such as myocardial infarction). The criteria for the transfusion of blood products were a hemoglobin level of < 8 g/dL or a hemoglobin level of < 10 g/dL in a patient with symptomatic anemia or deemed at high risk because of notable underlying cardiac comorbidities. Blood was administered 1 unit at a time, and the presence of symptoms or signs was reassessed.

### 2.7 Statistical analysis

Data were analyzed using the statistical software package SPSS version 25.0 (Chicago, IL). Continuous variables were described as the mean ± standard deviation, and differences between groups were analyzed using a series of one-way analysis of variance (ANOVA) with Bonferroni's post-hoc test, while differences between groups over time were analyzed using multi-way ANOVA with Bonferroni post-hoc test. Categorical variables were described as the number (%), and were analyzed by Fisher exact test. A *P* value of < .05 was considered statistically significant.

## 3 Discussion

TXA has gained popularity because of itsefficacy and ease of administration. Numerous studies have shown that intravenous TXA reduces perioperative blood loss and postoperative transfusion rates through its action as a potent antifibrinolytic.^[14,15]^ Despite several recent studies reporting the safety of intravenous TXA in spine surgery, there is still concern about its safety profile.^[16,17]^ Topical TXA has been utilized as an alternative; however, the efficacy and safety of topical TXA have not been well reported, as the majority of studies have been underpowered randomized clinical trials or retrospective in nature.^[18]^ Therefore, the goal of the present study was to perform an adequately powered, high-quality randomized clinical trial analyzing the efficacy and safety of both intravenous and topical TXA in lumbar interbody fusion, with an emphasis on perioperative calculated blood loss, total drain output at 24 hours, and perioperative blood transfusion rate.

There are several limitations to this study. We did not include a group of patients who did not receive TXA. From an ethical standpoint, it is reasonable to assert that the literature at this point would not support TXA versus no-TXA groups. Another potential limitation is that the study population contains heterogeneity such as varying patient diagnosis and surgical technique/approach. Despite these limitations, the validity of our results should be maintained, as the same methodology was applied to both treatment arms.

## Author contributions

**Conceptualization:** Fei Song.

**Data curation:** Fei Song.

**Formal analysis:** Fei Song.

**Funding acquisition:** Zhouhai Zheng.

**Investigation:** Fei Song, Zhouhai Zheng.

**Methodology:** Zhouhai Zheng.

**Resources:** Zhouhai Zheng.

**Software:** Zhouhai Zheng.

**Supervision:** Zhouhai Zheng.

**Validation:** Fei Song.

**Visualization:** Fei Song.

**Writing – original draft:** Fei Song, Zhouhai Zheng.

**Writing – review & editing:** Fei Song, Zhouhai Zheng.
